# Broadband Optical Mammography: Chromophore Concentration and Hemoglobin Saturation Contrast in Breast Cancer

**DOI:** 10.1371/journal.pone.0117322

**Published:** 2015-03-17

**Authors:** Pamela G. Anderson, Jana M. Kainerstorfer, Angelo Sassaroli, Nishanth Krishnamurthy, Marc J. Homer, Roger A. Graham, Sergio Fantini

**Affiliations:** 1 Department of Biomedical Engineering, Tufts University, 4 Colby Street, Medford, Massachusetts, 02155, United States of America; 2 Tufts Medical Center, Department of Radiology, 800 Washington Street, Boston, Massachusetts, 02111, United States of America; 3 Tufts Medical Center, Department of Surgery, 800 Washington Street, Boston, Massachusetts, 02111, United States of America; University of Pécs Medical School, HUNGARY

## Abstract

This study reports the optical characterization and quantitative oximetry of human breast cancer using spectrally-resolved images collected with a broadband, continuous-wave optical mammography instrument. On twenty-six cancer patients, we collected two-dimensional optical mammograms and created maps of the concentrations of hemoglobin, water, and lipids, as well as the oxygen saturation of hemoglobin. For each cancerous breast, we analyzed the difference between the tumor region (as identified by x-ray and optical mammography) and the remainder of breast tissue. With respect to the surrounding tissue, we found that cancer regions have significantly higher concentrations of total hemoglobin (+2.4±0.4 μM) and water (+7±1% v/v), and significantly lower lipid concentration (8±2% v/v) and oxygen saturation of hemoglobin (5±1%). We also found a significant correlation between the tumor optical contrast and the grade of breast cancer as quantified by the Nottingham histologic score; this demonstrates how optical signatures may be representative of metabolic and morphological features, as well as the aggressive potential of the tumor.

## Introduction

Diffuse optical imaging with near-infrared spectroscopy (NIRS) uses light in the wavelength range of 600–1000 nm to image breast tissue. Optical mammography offers a number of benefits by realizing a safe, non-ionizing, cost-effective imaging modality. From the patient’s perspective, there are two distinct advantages of optical mammography over x-ray mammography: no radiation exposure and no pain from compression. Using optical imaging methods, breast maps can be created of the concentrations of deoxy-hemoglobin, oxy-hemoglobin, water, and lipids (denoted as [Hb], [HbO], [water], [lipid], respectively, where the square brackets designate concentration in tissue). When characterizing differences between breast tumors and healthy tissue, elevated levels of hemoglobin concentration have been consistently found in cancerous tissue [1–11]. This increase in hemoglobin concentration reflects the known occurrence of angiogenesis. Although a fewer number of studies have investigated the concentration of water and lipids in the breast, it has been found that tumors have greater water content [1,6,9–11] and lower lipid content [1,11] when compared to non-cancerous tissue.

This study focuses on an additional NIRS measured parameter, the oxygen saturation of hemoglobin, which is also referred to as tissue saturation and hemoglobin saturation in the diffuse optics field. Here, to maintain consistency, we indicate oxygen saturation of hemoglobin as hemoglobin saturation (SO_2_). SO_2_ is defined as the ratio of oxy-hemoglobin to total hemoglobin concentration ([HbO]/[HbT]) and reflects a balance between the amount of oxygen delivered by the circulation per unit time per unit volume of tissue, and the amount of oxygen diffused from the capillary bed to the tissue [12]. It is important to observe that SO_2_, as defined here, is an oxygenation measure that solely refers to the oxygen bound to hemoglobin. By contrast, tissue oxygenation, which is related to but different from SO_2_, is representative of the total oxygen content available for tissue cells to use as a source of metabolic energy. This oxygen can come from intracellular, interstitial and vascular compartments [13]. Since the intracellular and interstitial oxygen is not bound to hemoglobin, tissue oxygenation cannot be directly measured with NIRS methods. To directly measure tissue oxygenation, the gold standard is an Eppendorf pO_2_ histography system which uses microelectrodes to obtain the oxygen partial pressure (pO_2_) of tissue in mmHg [13]. These two parameters, SO_2_ and pO_2_, are of interest in cancer characterization because they serve as indicators of the metabolic activity in malignant tumors. In the next paragraph, we provide a description of the tissue oxygen content, in terms of SO_2_ and pO_2_, on the basis of tumor microenvironment studies.

Understanding how SO_2_ and pO_2_ change in tumors using direct, high spatial resolution measurement techniques provides insight when interpreting SO_2_ values in breast cancer found from lower resolution methods such as diffuse optical imaging. Low tissue oxygenation, or hypoxia, has been reported in human tumors for decades [14–16]. Hypoxia occurs when there is a reduced amount of oxygen available which limits normal cellular functions. This hypoxic condition is due to the tumor’s increased metabolic demand in combination with the heterogeneously distributed perfusion from the chaotic, tortuous, leaky tumor blood vessels formed during angiogenesis [14,17]. The pO_2_ in tumors has been measured directly using invasive polarographic microelectrodes where lower pO_2_ has been found in breast cancer compared to healthy tissue [15]. An inverse relationship was reported between pO_2_ and tumor perfusion; poorly perfused tumors had a greater amount of tissue hypoxia, whereas well perfused tumors had a lesser amount of tissue hypoxia [18]. To quantify the oxygen in just the vascular compartment (SO_2_) of the microenvironment of a tumor, cryospectrophotometric methods have been used. This process examines the red blood cells in the microvessels of frozen tissue slices and finds the concentration of deoxy-hemoglobin and oxy-hemoglobin using the optical absorption of these chromophores. A cryospectrophotometric study found decreased SO_2_ in excised oral cavity tumors, and, similar to pO_2_, the decrease of SO_2_ was dependent upon the level of vascularization [19]. In addition to a lower average SO_2_ in tumors compared to healthy tissue, a spatially heterogeneous distribution of SO_2_ was noted throughout the tumor [19]. Experiments have also been performed to simultaneously examine tissue hypoxia and SO_2_ in the microenvironment as a tumor develops. Sorg *et al*. non-invasively examined the hypoxic state of a mammary carcinoma tumor using fluorescent markers. They measured the SO_2_ based on the light transmission spectra through the tumor in the dorsal skin flap of a rat [20]. As the tumor grew over time, the cancerous tissue became more hypoxic and, as a result of angiogenesis, new microvessels were created where lower SO_2_ values were observed. Overtime, a heterogeneous distribution of SO_2_ was also found [20]. Microenvironment techniques measure lower mean values of SO_2_ and pO_2_ in tumors compared to healthy tissue [14–16,18,19].

An inconsistency in NIRS investigations remains in regards to how SO_2_ in breast cancer compares to SO_2_ in healthy tissue. Some studies have reported decreased SO_2_ within tumors [7, 9–10, 21] whereas others have found no significant difference in the SO_2_ of cancerous and healthy tissue [1,2,5,6,8,11]. Several reasons have been hypothesized as to why SO_2_ may not be a robust NIRS cancer biomarker. One possibility is the fact that different cancer types, stages, and locations may result in different SO_2_ contrasts measured with NIRS. Each of these factors, which differ across studies, may contribute to an inconsistent trend in how SO_2_ in all cancers compares to healthy tissue [2,11]. A different argument, based on the observation that in some studies the total hemoglobin concentration correlates to the level of SO_2_ in tumors, points out that the tumor SO_2_ may be comparable to (or even greater than) that of healthy tissue when the oxygen supply exceeds the oxidative metabolic needs of the tumor [22]. In fact, tumor blood flow has been found to increase in cancer when measured with diffuse correlation spectroscopy [23]. However, this overall rise in blood flow to the tumor does not necessarily indicate uniform perfusion to prevent regions of decreased pO_2_ and lower SO_2_. Furthermore, the balance between oxygen supply and demand may vary across tumor types, stages, and for different morphological features of angiogenic blood vessels. It is important to consider how SO_2_ is measured in diffuse optical imaging studies to further investigate why discrepancies exist as to whether or not SO_2_ significantly changes in cancer.

Different forms of instrumentation and measurement techniques used for diffuse optical breast imaging could potentially contribute to the inconsistent trend in cancer SO_2_ findings. There are tradeoffs between information content available, spectral resolution, and spatial sampling rate in the various optical breast imaging systems. Time domain [5–8] and frequency domain systems [9–10] benefit from not relying on any wavelength dependent assumptions for the unique absorption spectra of chromophores and the scattering properties of tissue. However, these two domains of investigations often feature limited spectral information. One exception is the instrument developed by Taroni *et al*., which is a time domain system using Ti:sapphire and supercontinuum fiber lasers to allow for extensive spectral sampling [24]. Continuous wave (CW) systems [21] are less complex and can measure a broadband spectrum, but are hindered by the inability to separate absorption from scattering [25]. Some instruments also use a combination of these domains, such as continuous wave sources along with laser diodes in the frequency domain [1,2,11]. Spatial configurations also differ over various NIRS systems, which may feature either transmission [2,5–9,11,21] or reflection [1,10] geometries. Different spatial sampling rates may also be employed, ranging from every 1 mm [8] to every 10 mm [1] or more. In this study, our instrument uses a broadband continuous wave spectrum, a high spatial sampling (every 2 mm) and a transmission geometry to exploit a novel combination of NIRS imaging features in an effort to investigate tumor-induced changes in SO_2_.

Apart from how optical breast data is collected, one should also consider the multiple ways NIRS studies evaluate tumor hemoglobin saturation and how the post-processing steps are applied for SO_2_ analysis. The selection of the reference tissue as the healthy control to which the tumor region is compared against is not consistent across studies. The reference tissue varies from: a) the average global background (excluding the tumor) of the same breast [2,5,7,9,11] to; b) a localized area of healthy tissue in the cancerous breast [5,6,8] to; c) the mirror region in the contralateral breast [1,10]. One study reported that regardless of the tissue reference selection the discrimination between tumor and healthy tissue remains the same [26]. However, depending on the tumor size and the heterogeneity of the healthy tissue, the choice of reference area may impact the level of SO_2_ contrast. It is also relevant to consider how a significant difference in the SO_2_ is determined between tumor and healthy tissue. Group comparisons have been made by averaging breast tumor properties across multiple patients and evaluating these properties against the averages across the reference tissues [1,6,7,26]. This form of analysis has the potential to establish a cutoff value that may differentiate cancer from healthy tissue. Relative changes have also been investigated, where the averages across the cancer region were normalized to the corresponding reference tissue and the contrast was examined [2,6,11]. Relative values take into consideration the fact that there may be patient-to-patient variations and require a reference measurement. In our work, we chose to compare the breast tumor properties to the global background within the same breast (with the exclusion of the tumor region) to find the relative difference in the tumor compared to the surrounding healthy tissue.

In addition to investigating SO_2_ in breast cancer, this work also focuses on our system’s ability to characterize breast tumors (in terms of chromophore concentrations and hemoglobin saturation) to determine the optical signature of cancer for therapy monitoring purposes in the future. Optical mammography holds promise as an imaging modality that is able to follow a patient’s response to neoadjuvant therapy [27–31]. Provided that the cancer can be located and monitored accurately, patients can be imaged throughout their neoadjuvant therapy at frequent time points in a safe, non-invasive, and cost-effective way using CW methods. In this work, we used a system in parallel plate, transmission geometry that acquires data every 2 mm to create chromophore maps of the breast. This study does not have any diagnostic aims and focuses on characterizing breast tumors using *a priori* information about the cancer location. We have examined 26 cancer cases and report the difference in [HbO], [Hb], [HbT], [water], [lipid], and SO_2_ between the tumor and background healthy tissue.

## Methods

### Experimental setup

**Fig 1:**
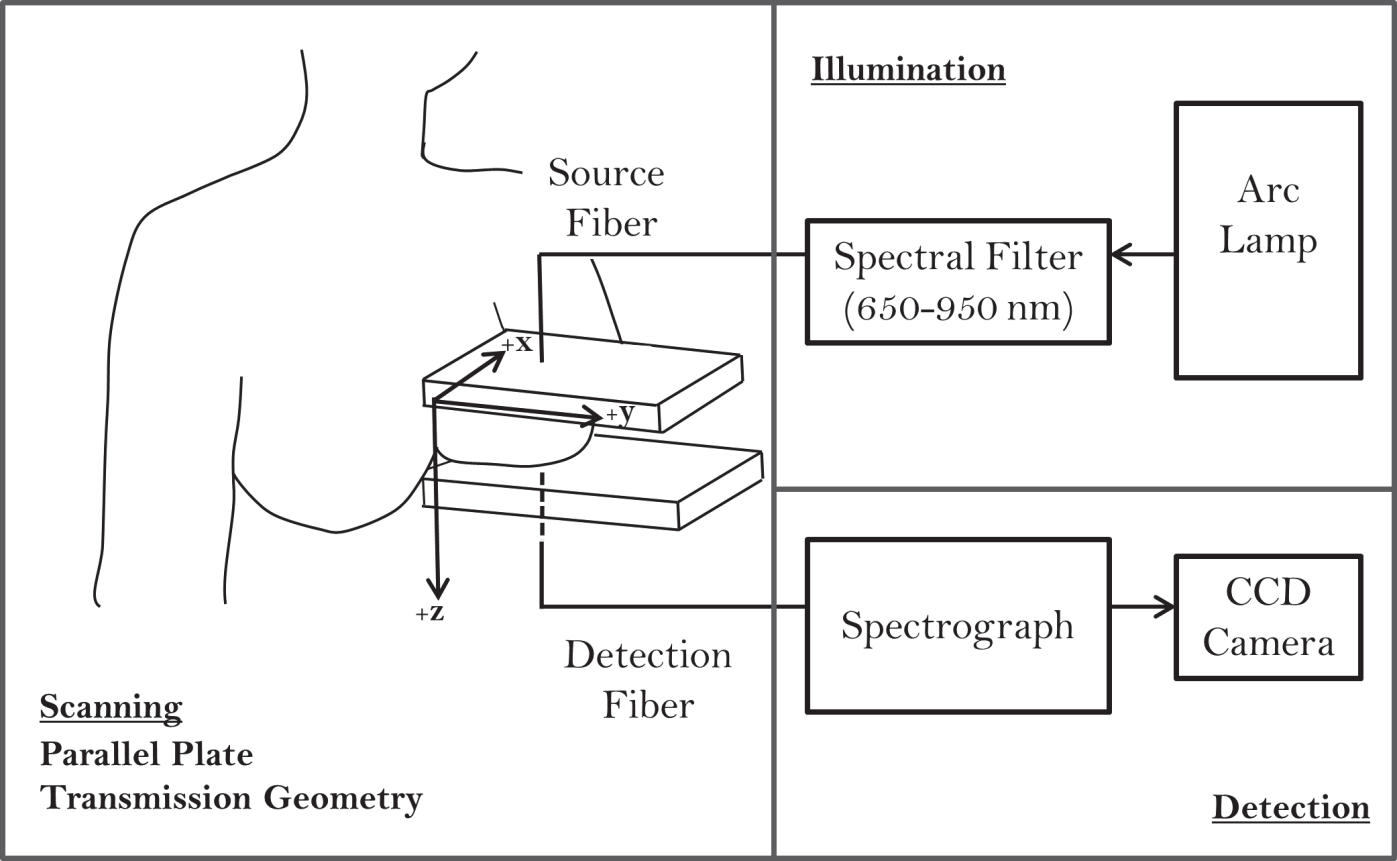
Schematic of the optical mammography instrument. The breast is imaged in a parallel plate, transmission geometry where source and detector fibers scan collinearly. The illumination scheme depicts the arc lamp which emits broadband, continuous wave light, filtered between 650 and 950 nm to the surface of the breast. The detected spectra are measured with a spectrograph and cooled CCD camera.

We have developed a broadband continuous-wave optical mammography system described in detail elsewhere [21]. A diagram of the optical mammography instrument is shown in Fig. 1. Imaging of the breast is performed in a parallel plate geometry where the breast is mildly compressed between two polycarbonate plates. This mild compression is intended to maintain tissue stability during the scan while also ensuring that the patient experiences no discomfort. The plate separation distance, corresponding to the maximum breast thickness in our measurement geometry, was recorded for each patient and the thickness ranges are reported in Table 1. A broadband light source (Xenon arc lamp, Model No. 6258, Newport Corporation, Irvine, CA), with spectral filters in place to eliminate ultraviolet (below 400 nm) and infrared (above 950 nm) wavelengths, is used to illuminate the breast tissue. Illumination and detection optical fibers (3 and 4 mm in diameter, respectively) scan collinearly, and data are captured every 2 mm in the *x*- and *y*- directions of the plates (see Fig. 1 for coordinate system). Transmitted light is measured with a spectrograph (Model No. SP-150, Princeton Instruments, Acton, MA) and a cooled CCD camera (Model No. DU420A-BR_DD, Andor Technology, South Windsor, CT) with a 45 ms exposure time and a 12 ms readout time. Spectral data is acquired from 650 nm to 950 nm, with an 8 nm wavelength resolution.

**Table 1:** Patient Demographics and relevant clinical information.

		Nottingham Histologic Score					
		4	5	6	7	8	No Score Available
**Cancer Cases**	26	1	4	11	3	2	5
**Age (years)**							
Average ± std	57 ± 11						
	36–45			1			3
	46–55		2	5			1
	56–65	1	1	1	1	1	1
	66–76		1	4	2	1	
**Tumor Size (cm)**							
Average ± std	2.0 ± 1.4						
	0.5–0.9	1	2	3			
	1.0–1.9		1	6	2	1	1
	2.0–2.8		1		1		2
	3.0–5.5			2		1	2
**Cancer Type**								
	DCIS						3
	IDC			3	1		2
	IDC/DCIS	1	2	5	2	2	
	IDC/DCIS/LCIS		1	1			
	ILC			1			
	ILC/LCIS		1	1			
**Max. Breast Thickness (cm)**							
Average ± std	6.7 ± 1.1						
	4.3–5.5			2	2		1
	5.6–6.5			1		1	1
	6.6–7.5	1	2	6	1	1	2
	7.6–9.2		2	2			1

### Breast cancer patients and ethics statement

A total of 29 breast cancer patients were imaged during this study. Three of the patient data sets were not included in the analysis (two cases were excluded due to technical problems with the optical imaging system and one case because the cancer was located outside of the imaged area) leaving 26 patient datasets for investigation. The associated patient demographics and relevant clinical information are shown in Table 1, including the patient age, cancer size and type, and maximum breast thickness. The cancer size was taken as the maximum dimension measured by the radiologist from the x-ray mammogram. The table categorizes the number of patients within given ranges for each of these parameters by the Nottingham histologic score. This score is a histopathologic measure of the aggressiveness of breast cancer based on 3 parameters: the percent of tubule formation, the mitotic count, and the nuclear pleomorphism of the tumor. Each of these parameters is then given a score from 1–3, and the scores are then added together to create integer values between 3 and 9. Scores of 3–5 are defined as low grade (grade 1), 4–7 as intermediate grade (grade 2), and 8–9 as high grade (grade 3) [32]. The Nottingham histologic score was not available for 5 of the 26 patients, as 3 of the patients had ductal carcinoma in situ only and 2 of the patients with invasive ductal carcinoma were diagnosed by needle biopsy and have not had their definitive surgery and final pathology. Patients were recruited and imaged either before biopsy, based on suspicious findings on the x-ray mammogram (n = 13), or after biopsy (n = 13). Patients who had a biopsy before the optical mammogram were imaged at least one week after the invasive procedure, which was reported to be an adequate amount of time to wait in order for there to be no significant differences in the contrast of the optical parameters when compared to patients who were imaged before biopsy [2]. To ensure there was no difference between the two groups, our analysis was also performed separately on the patients who received a biopsy before their optical mammogram and the patients who had their biopsy after the optical mammogram to determine if the results differed between the two groups. Imaging was performed on both breasts of the patients using a cranio-caudal view (axial plane). The scan time was dependent on the breast size and varied between 4 and 10 minutes. The study was approved by the Institutional Review Board at Tufts Medical Center. Each recruited patient read and signed an informed consent form before participating in the study.

### Continuous-wave diffusion model

A continuous wave photon migration model was used to process the breast transmission data. The model, based on work by Contini *et al*., uses the solution of the diffusion equation in slab geometry [33]. A point source and extrapolated boundary conditions have been applied, where the transmittance *T* (defined as the ratio of the transmitted intensity to the incident power) at a wavelength λ, is given by [33]:

$$\begin{array}{c} T\left(\lambda \right)=\frac{K}{4\pi }\left(\frac{z_{1,m}\left(\lambda \right)}{{\left(z_{1,m}{}^{2}\left(\lambda \right)\right)}^{3/2}}\right)\left\{1+\sqrt{3\mu_{a}\left(\lambda \right)\mu_{s}^{\prime }\left(\lambda \right)z_{1,m}{}^{2}\left(\lambda \right)}\right\}\text{exp}\left\{-\sqrt{3\mu_{a}\left(\lambda \right)\mu_{s}^{\prime }\left(\lambda \right)z_{1,m}{}^{2}\left(\lambda \right)}\right\}\\ -\left(\frac{z_{2,m}\left(\lambda \right)}{{\left(z_{2,m}{}^{2}\left(\lambda \right)\right)}^{3/2}}\right)\left\{1+\sqrt{3\mu_{a}\left(\lambda \right)\mu_{s}^{\prime }\left(\lambda \right)z_{2,m}{}^{2}\left(\lambda \right)}\right\}\text{exp}\left\{-\sqrt{3\mu_{a}\left(\lambda \right)\mu_{s}^{\prime }\left(\lambda \right)z_{2,m}{}^{2}\left(\lambda \right)}\right\} \end{array}$$ (1)

where *K* is a dimensionless scaling parameter, μ_*a*_(λ) is the wavelength dependent absorption coefficient, μ_s_ˈ(λ) is the wavelength dependent reduced scattering coefficient and *z*_*1*,*m*_ and *z*_*2*,*m*_ are coefficients of order *m* (*m* = 0, ±1, ±2…), given as:

$$z_{1,m}=s\left(1-2m\right)-4mz_{e}-z_{0}$$ (2)

$$z_{2,m}=s\left(1-2m\right)-\left(4m-2\right)z_{e}+z_{0}$$ (3)

where *s* is the tissue thickness, *z*_*0*_ = 1/μ_s_ˈ, is the depth of the effective point-source location within tissue, and *z = z*_*e*_ is the plane that defines the extrapolated boundary. In our model we used orders up to |*m|* = 6 which yield a truncation error less than 0.1% [33]. The *z*-coordinate of the extrapolated boundary is defined by [33]:

$$z_{e}\left(\lambda \right)=\frac{2A}{3\mu_{s}^{\prime }\left(\lambda \right)}$$ (4)

where the reflection parameter *A* is defined in terms of integrals over the incidence angle θ_*i*_ (between the incoming photons and the normal to the tissue slab interface) and the Fresnel reflection coefficient *R*(θ_*i*_) [33]:

$$A=\frac{1+3\int_{0}^{\pi /2}R\left(\theta_{i}\right){\text{cos}}^{2}\left(\theta_{i}\right)\text{sin}\left(\theta_{i}\right)d\theta_{i}}{1-2\int_{0}^{\pi /2}R\left(\theta_{i}\right)\text{cos}\left(\theta_{i}\right)\text{sin}\left(\theta_{i}\right)d\theta_{i}}$$ (5)

The Fresnel reflection coefficient represents the fraction of reflected photons at the tissue interface, and it depends on the incidence angle θ_*i*_ and the indices of refractions of the external medium (*n*_air_) and diffusive medium (*n*_tissue_), which we set at 1.0 and 1.4, respectively. As a result, the reflection parameter took the value *A* = 2.9, which we kept constant in our calculations. The external medium could also be considered as polycarbonate, where *n*_ext_ = 1.58, which would be a suitable value to use in the *A* parameter calculation where the breast is in contact with the plates. Although the choice of the external index of refraction changes the magnitude of the *A* parameter, the differences in chromophore concentration between the tumor and surrounding tissue reported in the results section are minimally impacted (to within the standard deviation of the data). The equations for the wavelength-dependent absorption coefficient (μ_*a*_) and wavelength-dependent reduced scattering coefficient (μ_*s*_ˈ), are given by:

$$\mu_{a}\left(\lambda \right)=\varepsilon_{\text{HbO}}\left[\text{HbO}\right]+\varepsilon_{\text{Hb}}\left[\text{Hb}\right]+\varepsilon_{\text{water}}\left[\text{water}\right]+\varepsilon_{\text{lipid}}\left[\text{lipid}\right]$$ (6)

$$\mu_{s}^{\prime }\left(\lambda \right)=\mu_{s}^{\prime }\left(\lambda_{0}\right){\left(\frac{\lambda }{\lambda_{0}}\right)}^{-b}$$ (7)

The absorption coefficient is dependent upon [HbO], [Hb], [water], [lipid], and their corresponding extinction coefficients (ε_HbO_, ε_Hb_, ε_water_, ε_lipid_). Equation (7) introduces a reference wavelength, λ_0_ (which is 650 nm in this work) and a scattering power, *b*, to define the wavelength dependence of the reduced scattering coefficient.

Equation (1) is used as the forward solver in an inversion procedure based on the Levenberg-Marquardt method [34]. This inversion procedure directly solves for the concentrations of the chromophores by using Eq. (6) in place of μ_*a*_ in Eq. (1). The variable *K* in Eq. (1) is used as a scaling factor to adjust the model-calculated transmittance spectra (expressed in mm^−2^) in order to match the measured transmittance spectra (expressed in arbitrary units). We first treated *K* as a variable which was fit separately for the right and left breast of each patient. Then *K* was fixed as the average *K* value between the right and left breast scans, and the final fits were performed to find the chromophore concentrations in the breast.

The measured breast transmission spectra, *T*(λ), were used as the input to the inversion procedure. Due to the strong spectral features from the Xenon arc lamp, a reference spectrum was measured in order to remove these features from patients’ transmission data. The reference spectrum was obtained through air after placing neutral density filters at the source fiber end. The transmission spectra from the patient scans were then divided by this reference spectrum to suppress the features of the Xenon arc lamp. Although data in the wavelength range of 650–950 nm were collected, due to the low signal measured at the longer wavelengths (above 850 nm) the reference spectrum was not always able to remove these Xenon spectral signatures. For this reason, we did not use data beyond 850 nm when performing fits with the inverse model. However, the relative intensity of the light at these higher wavelengths still provided useful information for tissue thickness estimation (see the following paragraphs and Eq. (8)). Since absorption and scattering contributions cannot be separated with continuous-wave (CW) data [25], we set the scattering parameters (μ_s_ˈ(λ_0_) and *b*) to values found in the literature (namely, μ_s_ˈ(650 nm) = 10.8 cm^−1^ and *b* = 1.0) [5] and assumed them to be constant throughout the entire breast. By fixing these parameters, the stability and robustness of the inverse model was improved compared to when the fit was finding the chromophore concentrations and scattering parameters. The starting values for the chromophore concentrations (the parameters being fit for) were re-initialized to the same values for each pixel of breast data and the fit was performed. The fitting process was carried out on a computer with 3.49 GB of RAM and a 3.20 GHz single core Intel Pentium 4 processor. Depending on the number of pixels collected in a scan, the fitting procedure for one breast dataset, where six maps were produced, took one to three minutes. Although we create six maps, only [Hb], [HbO], [water], and [lipid] are independent ([HbT] and [SO_2_] maps are derived from [Hb] and [HbO] maps).

**Fig 2:**
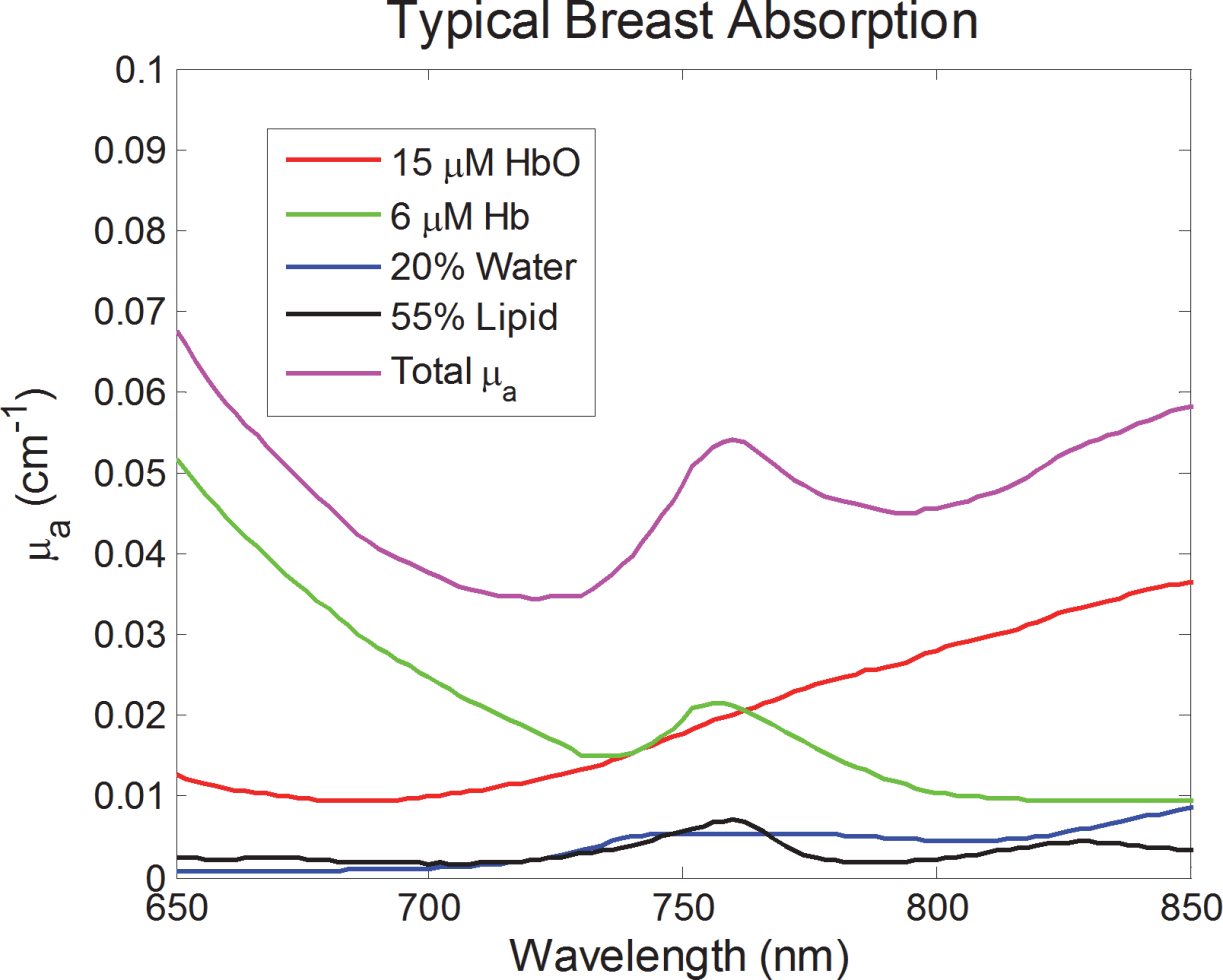
Absorption spectra for typical concentrations of the four main chromophores in breast tissue and the resulting total absorption coefficient. The different shapes of each chromophore’s absorption spectrum portray why we can recover unique concentration of deoxy-hemoglobin, oxy-hemoglobin, water, and lipid.

By using a broadband continuous wave system at wavelengths 650–850 nm, our inverse model was able to recover the volume percentages (% v/v) of water and lipids in the breast. Although other instruments often use longer wavelengths (sometimes including the water and lipid absorption peaks at ∼970 and ∼930 nm, respectively), we are able to capitalize on our broadband signal and still solve for all four chromophores on the basis of their different absorption spectral shapes. Fig. 2 shows the absorption spectra of each chromophore and the total absorption spectrum for typical breast tissue over our wavelength range. To verify the accuracy of this method to recover hemoglobin concentration, water content, and lipid content, we computed data by using Eq. (1) (*K* = 1, [Hb] = 6 μM, [HbO] = 15 μM, [water] = 20%, [lipids] = 55%) with 5% random noise added to the transmission spectra and used it as an input into our inversion procedure. The chromophore concentrations retrieved by the inverse model were within 5% of the actual values, which gave us confidence that our data analysis scheme was accurate.

Another input into the model was the tissue thickness at each pixel. If the breast shape, and associated tissue thickness variability, was not taken into account, we found that the chromophores concentration maps contained a breast thickness bias. Previously proposed methods for estimating breast tissue thickness have used frequency domain [35] or time domain [36] information and were not applicable here. To get an estimate of the breast thickness throughout the scanned area using only our continuous wave data, we started by defining a “reference point” as the pixel at the center of the breast, closest to the chest wall. The tissue thickness at the reference point, *s*_*0*_, corresponds to the plate separation (which ranged between 4.3 and 9.2 cm). The transmission spectrum at this reference pixel was then used as the input into the inversion procedure (described above) and the chromophore concentrations were determined. The effective attenuation coefficient $\left(\mu_{\text{eff}}=\sqrt{3\mu_{a}\mu_{s}^{\prime }}\right)$ at the reference point was then obtained. In order to estimate the breast thickness at each pixel we made two assumptions: a) the breast is a homogeneous medium, therefore the optical properties at each pixel are the same as those calculated at the “reference point”, and b) the ratio of intensities detected at two different pixels (characterized by different breast thicknesses) can be approximated by the ratio of detected intensities at two different source-detector separations in an infinite medium geometry. Although these two assumptions are only rough approximations, they rely on changes in transmitted intensity being dominantly affected by variations in tissue thickness, rather than spatial variations in the optical properties or boundary conditions associated with the breast geometry. An optimal wavelength (λ*) was identified where the variation in μ_eff_ is smallest across a wide range of breast optical properties and by using the transmitted intensity at λ*, inhomogeneities within a breast do not play a large role in our thickness estimation. Using the solution of the diffusion equation in the infinite medium geometry, the ratio of intensities (*I*, *I*_0_) detected at this optimal wavelength at two different tissue thicknesses (*s*, *s*_0_, respectively) is given by:

$$\frac{I\left(\lambda^{*}\right)}{I_{0}\left(\lambda^{*}\right)}=\frac{\left(s_{0}\right)e^{\mu_{\text{eff}}\left(\lambda^{*}\right)s_{0}}}{\left(s\right)e^{\mu_{\text{eff}}\left(\lambda^{*}\right)s}}$$ (8)

The ratio *I*(λ*)/*I*_0_(λ*) for an infinite geometry in Eq. (8), is approximated by the ratio *T*(λ*)/*T*_0_(λ*) in the slab geometry of interest here. The transmitted intensity at the optimal wavelength and at the reference point in the breast scan (characterized by the thickness *s*_0_) is given as *T*_0_(λ*), while *T*(λ*) and *s* are the transmitted intensity and tissue thickness, respectively, at the general pixel. The tissue thickness, *s*, was calculated at every pixel by solving the transcendental Eq. (8) with the *fzero* function in Matlab (MathWorks, Natick, MA).

**Fig 3:**
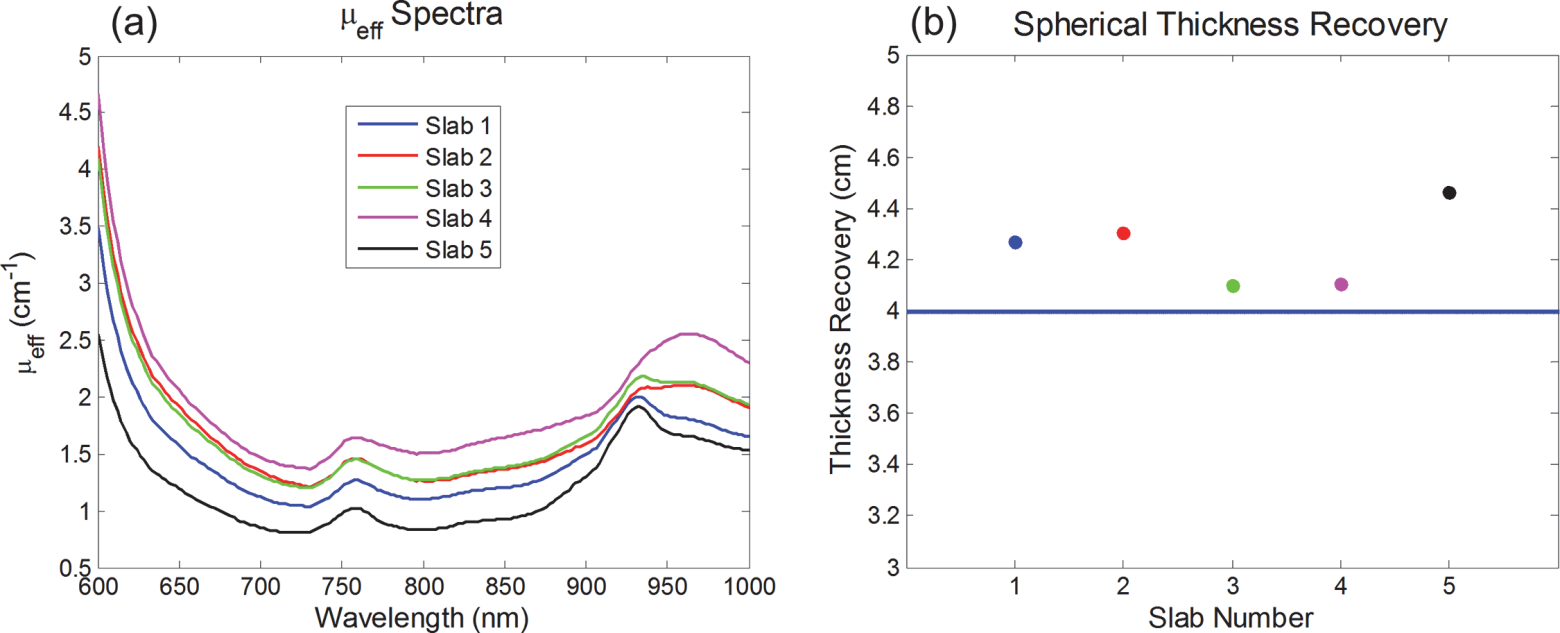
Computed μ_eff_ spectra and spherical geometry thickness recovery data. (a) Five computed μ_eff_ spectra for different chromophore concentrations that span the range in breast tissue from the literature (each slab’s chromophore concentrations are listed below). The μ_eff_ variability is minimal around 925 nm. (b) Thickness recovered for the 4 cm chord distance in a spherical medium with the same chromophore concentrations as in Slab 1. The reference transmittance, *T*_0_(λ*), for the application of Eq. (8) is taken to be the one for a 5 cm thick infinite slab with the chromophore concentrations of Slabs 1–5, as indicated in the *x* axis. Chromophore concentrations of each slab are listed as ([HbO], [Hb], [water], [lipid]): Slab 1 (14uM, 7uM, 20%, 65%); Slab 2 (17uM, 11uM, 30%, 45%); Slab 3 (18uM, 10uM, 30%, 60%); Slab 4 (28uM, 12uM, 45%, 30%); Slab 5 (6uM, 4uM, 20%, 75%).

The error due to the first assumption, *i*.*e*, that the μ_eff_ is spatially homogeneous, is wavelength dependent. The variation in the magnitude of μ_eff_ can be observed in Fig. 3(A), which reports the effective attenuation coefficient for five different sets of [HbO], [Hb], [water], and [lipid] that span the range found in the literature for breast tissue [12]. The scattering properties were set to be the same for all computed data. The optimal wavelength identified as λ* above was found where the variability in the magnitude of μ_eff_ is smallest, which is desired when determining the wavelength used in our thickness recovery method. We found this optimal wavelength to be λ* ∼ 925 nm. We tested our method by using Eq. (1) to compute five transmittance curves in a slab geometry (referred to as slabs 1, 2, 3, 4 and 5) with the same set of [HbO], [Hb], [water], and [lipid] as described in Fig. 3 and with source detector separations equal to 5 cm. These five curves represent different baseline *T*_0_. To mimic the collection of light at the boundary of the breast, we computed transmittance data in a spherical geometry with a diameter equal to the slab thickness, namely 5 cm, and the chromophore concentrations of slab 1 (given in the caption of Fig. 3). This spectrum was found by using the solution of the diffusion equation in the spherical geometry with extrapolated boundary conditions [37]. Spherical transmittance data were generated at a direct source-detector separation of 4 cm (which is the local chord length shorter than the sphere diameter of 5 cm) and this 4 cm represents the thickness to be recovered. The spherical geometry transmittance at λ*, *T*(λ*), was used in Eq. (8) to find the thickness *s*, the unknown of our problem (4 cm in this case). We performed five different thickness retrieval tests, for *T*_0_(λ*), by using the five sets of slab transmittance data associated with the five chromophore concentration cases in Fig. 3. This range of chromophore concentrations at the reference pixel specifies the error in thickness recovery when the optical properties of the reference and general pixel are the same (reference: slab 1) or different (reference: slabs 2–5). These computations served to test our method based on Eq. (8) under known conditions. Additionally, measurements have been performed on a homogeneous, breast-shaped, silicone, tissue-mimicking phantom to experimentally validate the tissue thickness estimation algorithm. This phantom had known absorption and scattering properties (μ_*a*_(690nm) = 0.04 cm^−1^ and μ_s_ˈ(690nm) = 4.9 cm^−1^) and had edges that curved as a semi-circle with a 3.0 cm radius.

### Tumor localization

We studied 26 breast cancer patients and analyzed the corresponding breast maps of the spatial distributions of [HbO], [Hb], [HbT], [water], [lipid], and SO_2_. The identification of tumor pixels in the optical images was done using the map of deoxy-hemoglobin concentration, based on a report that [Hb] is highly sensitive to breast tumors [1]. First, a broad tumor region was determined from the known tumor location on the x-ray mammograms and the morphological similarity of the optical and x-ray breast images in the cranio-caudal view. Because the level of compression and exact breast placement differ between the optical and x-ray imaging modalities, an exact geometrical co-registration was not possible. Furthermore, the varying sources of contrast between x-ray images (tissue density and morphology, microcalcifications, etc.) and optical images ([HbT], [water], [lipid], SO_2_, tissue scattering, etc.) and the spatial resolution differences between the two imaging modalities may account for a different size and precise location of breast tumors in the images generated by the two techniques. Therefore, we only used the corresponding x-ray image to guide the placement of a rectangular area to where we would then identify the tumor region in the optical images. The pixel having the maximum [Hb] within this rectangular tumor region was found and all the pixels, within this same region, with [Hb] of at least 75% of the maximal concentration were assigned to the tumor region of interest (ROI). The choice of a 75% threshold was found to retain a relatively large number of pixels for the tumor ROI.

In order to characterize the difference between tissue properties in tumors and in surrounding healthy tissue, we first found the average value for each chromophore over the identified tumor ROI. Then the average background was determined by taking the mean over all the pixels outside of the initially identified rectangular tumor area. The differences between the average values in the tumor and background tissue were then considered. The contralateral (healthy) breasts for each cancer patient were used as controls for this study to determine if any significant changes were observed in the mirrored tumor location compared to the corresponding background tissue. The contralateral calculations were performed by reflecting the tumor rectangular area and the corresponding ROI of the cancerous breast onto the healthy breast to identify a region in the healthy breast that is symmetrical to the tumor ROI in the cancerous breast. The average over the background pixels (determined the same way as in the cancerous breast) was then found in the contralateral breast and the difference in chromophore concentrations between the tumor ROI and background tissue were calculated.

### Statistical analysis

To determine the significance of the difference between the measured quantities at the tumor location and the background tissue, we carried out a group analysis over the 26 subjects by using the Wilcoxon signed rank test. A non-parametric test was well suited in this case where the nature of the statistical distribution of the results, in particular its Gaussianity, is unknown. The signed rank test was used to determine whether the means of the chromophore concentrations within the tumor ROI were higher or lower than the average of the global background within that same breast. All the tumor ROI means were paired with their corresponding background means and one-tailed tests were performed with a significance level of 0.05. This test was also used to determine if any significance was found between the symmetrical tumor ROI in the contralateral breast and the corresponding background tissue. This contralateral comparison was carried out to ensure that there was no bias in the cancerous breast data (possibly from geometrical artifacts due to the breast thickness variability and deviations from the assumed slab geometry as the optical scanner approaches the edge of the breast projection). The contralateral test also served as a control in the statistical calculations when comparing the significance level of the signed rank test. All of the statistical calculations were performed in R software.

## Results

### Optical estimation of tissue thickness: tests with computed data and phantom measurements

The results of our thickness recovery computations [using Eq. (8)] for the five different *T*_0_(λ*) and the spherical geometry transmittance *T*(λ*) are shown in Fig. 3(B). It can be seen that the thickness recovery always fell within 0.5 cm of the correct value (4 cm). Despite the low optical signal at 925 nm for patient data, we found that the relative insensitivity of μ_eff_ to optical heterogeneities at this wavelength results in a robust method for optical estimation of breast tissue thickness [see Fig. 3(A)]. At these wavelengths (around 925 nm) there is a higher absorption dominated by water and lipid, where their corresponding extinction coefficients are close in magnitude. If there is a region of the breast where the chromophore concentrations differ from the reference point, the 925 nm range will experience less of an attenuation change compared to shorter wavelengths, where hemoglobin is the dominant absorber. Experimental thickness estimation results were also found after scanning the homogeneous, breast-shaped, silicone phantom with our optical mammography instrument. Using the recovered thickness as an input to the diffusion based model (described in the methods section), we found the absorption coefficient throughout the phantom and calculated the percent error from the known absorption for each pixel. The average percent error on the absorption coefficient across the entire phantom was 12%, which indicates the homogeneity of the phantom and accuracy of the thickness estimation algorithm.

### Breast Maps

**Fig 4:**
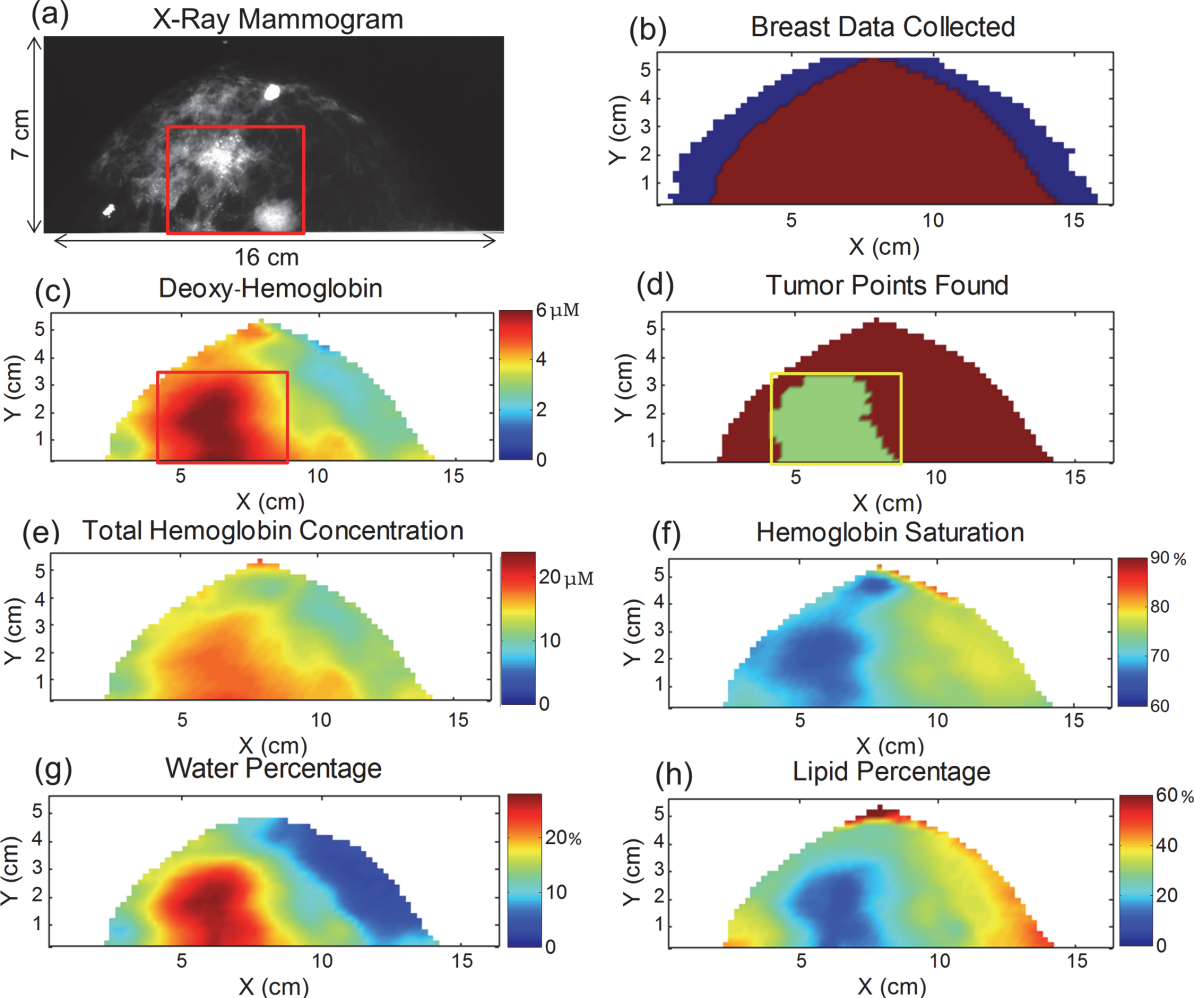
Maps for a 72 year old patient who has invasive ductal carcinoma with ductal carcinoma in situ in her left breast. (a) X-ray mammogram where two foci of cancer spanning 3.9 cm in combined maximum diameter are located in the 5 and 6 o’clock positions within the breast (shown in the red box). (b) The data discarded at the sides of the scanned breast are depicted in blue. (c) The deoxy-hemoglobin map is used to located the tumor ROI within the red rectangular area (based on the x-ray mammogram as described in the methods section) (d) The tumor rectangular box is shown in yellow and the shaded green region represents the tumor ROI, consisting of pixels with [Hb] to within 75% of the maximum [Hb] in the box. Maps of (e) total hemoglobin concentration, (f) hemoglobin saturation, (g) water percentage, and (h) lipid percentage.

**Fig 5:**
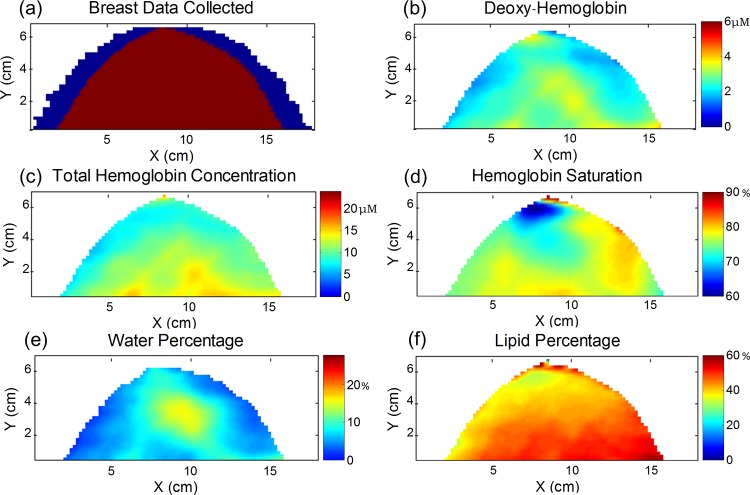
Optical maps of the healthy contralateral breast for the cancer patient shown in Fig. 4. (A) The data discarded at the sides of the scanned breast are shown in blue. Maps of the (b) deoxy-hemoglobin concentration (c) total hemoglobin concentration, (d) hemoglobin saturation, (e) water percentage, and (f) lipid percentage are shown on the same color scale as the maps in Fig. 4 (C, E-H).

Fig. 4 shows the maps for a 72 year old patient who has invasive ductal carcinoma with ductal carcinoma in situ in her left breast. The x-ray mammogram is shown in Fig. 4(A), where there are two foci of cancer that together span 3.9 cm in maximum dimension as reported by the radiologist. The map in Fig. 4(B) illustrates the optical data that was discarded due to edge effects close to the border of the breast (<20 mm in Fig. 4(B) and typically <20 mm in our study). The data along the far sides of the breast were discarded when the peak of the Xenon lamp (∼830 nm) saturated the 16-bit CCD camera. The tumor characterization process (described in the methods section) was then carried out on the [Hb] map where Fig. 4(C) shows the rectangular region that encloses the area of the tumor (as determined by x-ray mammography). The shaded pixels corresponding to the tumor ROI after the 75% threshold was applied can be seen in Fig. 4(D). Fig. 4(E)-(H) show the maps of [HbT], SO_2_, [water], and [lipid] in the breast. The corresponding contralateral healthy breast maps are given in Fig. 5(A)-(F) for this same cancer patient. The contralateral breast maps are given on the same color scale as the cancerous maps, illustrating the decreased level of contrast for the optical parameters in the healthy contralateral breast.

### Chromophore concentrations and hemoglobin saturation

**Fig 6:**
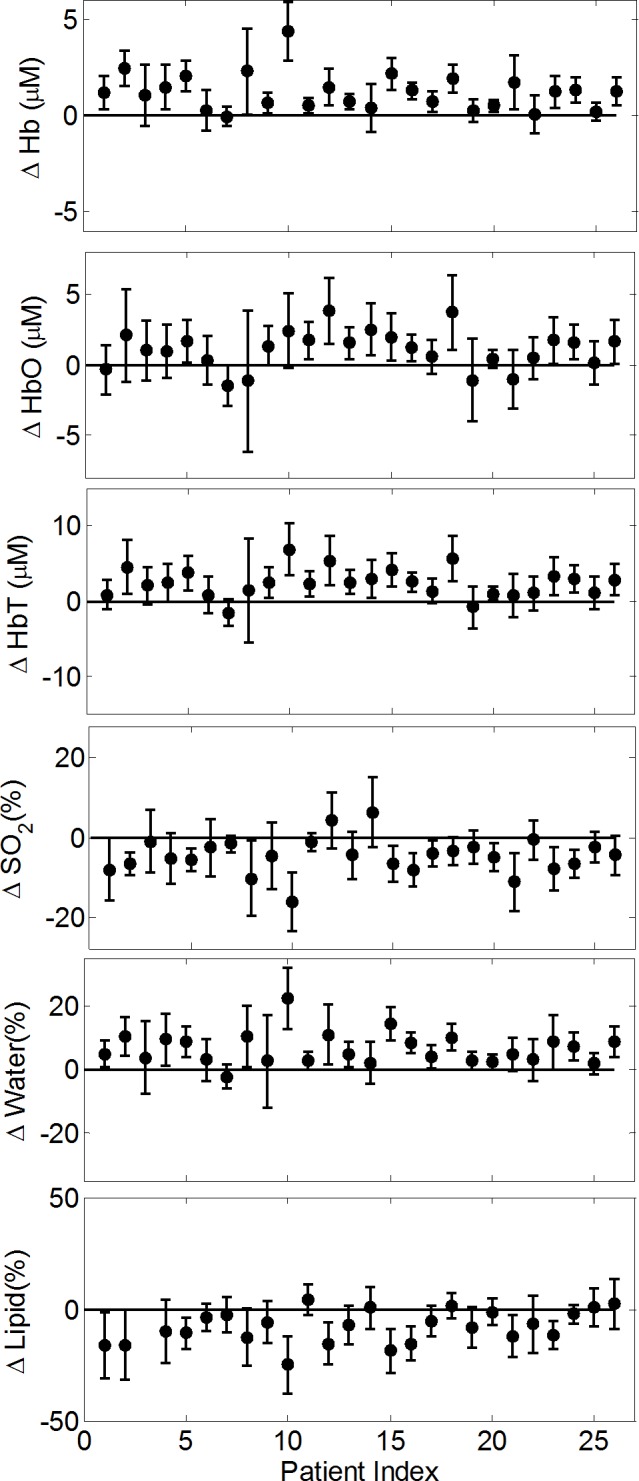
Individual difference quantities in the average concentration of deoxy-hemoglobin, oxy-hemoglobin, total hemoglobin, hemoglobin saturation, water concentration, and lipid concentration between the tumor region and background breast tissue for all 26 cancer patients. Each individual patient’s measurements are shown and error bars represent the standard deviation of the differences.

The differences between the average tumor ROI and background parameters for all cancer cases are shown in Fig. 6. These differences are referred to as the “difference parameters” for the remainder of the text. The error bars in these plots were calculated by taking the square root of the sum of the variances over the background and tumor ROI pixels, and represent the standard deviation of the difference. In the 26 cancer cases, the tumors featured an average increase of +1.1±0.3, +1.2±0.2 and +2.4±0.4 μM in [HbO], [Hb], and [HbT], respectively. The changes in hemoglobin concentrations were all found to be significant by the Wilcoxon signed rank test with *p* values less than 0.05. There was also an average increase of +7±1% in [water] and a decrease of −8±2% in [lipid] within the tumor region. The tumor SO_2_ was found to be on average −5±1% lower than the background tissue and only in 2 of the 26 cases did we not find the average SO_2_ of the tumor ROI to be lower than the background. The *p* values found for the [water], [lipid], and SO_2_ changes in tumors were all less than 0.05. The patients imaged before and after biopsy (n = 13 for each group) were also analyzed separately and significant changes (*p* < 0.05) between the tumor regions and surrounding tissue for all optical difference parameters were found for each group. Since our results do not change regardless of whether or not the patient underwent a biopsy over one week prior to the optical imaging, we kept all 26 patients grouped together for analysis. There was one case for a 36 year old patient (#3) where we did not include a data point in the lipid difference plot because the model found 0% [lipid] in the background and tumor regions, which is not physiologically reasonable. Table 2 shows the average of the difference parameters for chromophore concentrations and SO_2_, over the 26 patients, with the corresponding standard errors. The standard errors reported here indicate the variability across the 26 difference parameters found.

**Table 2:** Summary of the difference parameters averaged across the 26 cancer patients.

	Cancer Breast			Contralateral Breast		
	Average	Std. Err.	*p* Value	Average	Std. Err.	*p* Value
ΔHbO (μM)	+1.1	0.3	<0.01	−0.3	0.4	0.81
ΔHb (μM)	+1.2	0.2	<0.01	+0.1	0.1	0.35
ΔHbT (μM)	+2.4	0.4	<0.01	−0.2	0.5	0.71
ΔSO_2_ (%)	−5	1	<0.01	−1	1	0.11
ΔWater (%)	+7	1	<0.01	+1	1	0.10
ΔLipid (%)	−8	2	<0.01	−1	1	0.11

In 25 out of the 26 patients imaged, an increase in [water] was found in the tumor region. The difference between the [water] in the tumor and background tissue was found to be a more consistent feature of cancer compared to the [HbT] difference parameter in this study. We have found two patients (#7 and #19) where the [HbT] was not greater within the tumor when compared to the global background average (see Fig. 6). Patient #7 had a 0.6 cm tumor that was located by x-ray mammography in the upper outer quadrant at the level of the nipple (center of the breast). The plate separation distance for this patient was 6.5 cm, therefore we believe our inability to detect an increased level of [HbT] is due to the low optical contrast of this small cancer located deep inside the breast. As for the second case (patient #19), the region where the 0.7 cm tumor is located has an elevated total hemoglobin content with respect to the immediately surrounding area, but it is not significantly greater than the average taken over the background pixels.

Table 2 also reports the average of the difference parameters and standard errors for [HbO], [Hb], [HbT], [water], [lipid], and SO_2_ for the symmetrical tumor ROI and background tissue in the contralateral breast. Their *p* values are all above 0.05 showing a lack of significant difference between the symmetrical tumor ROI and background tissue in the contralateral breast. This result demonstrates that geometrical artifacts associated with the breast shape and boundary effects cannot account for the significant differences observed in the cancerous breasts. By comparing the results of the Wilcoxon signed rank test for the cancerous and contralateral breasts, the statistically significant findings for the cancerous breasts imply that the difference parameters for this group are significantly greater in magnitude compared to the contralateral, or control, difference parameters. To ensure this point, we also explicitly compared the difference parameters of the cancerous and contralateral breasts using the Wilcoxon signed rank test. The *p* values were all found to be less than 0.05, proving that the magnitude of the difference parameters in the cancerous breasts are significantly greater than in the contralateral breasts.

### Correlation between optically measured quantities and Nottingham histologic score

**Fig 7:**
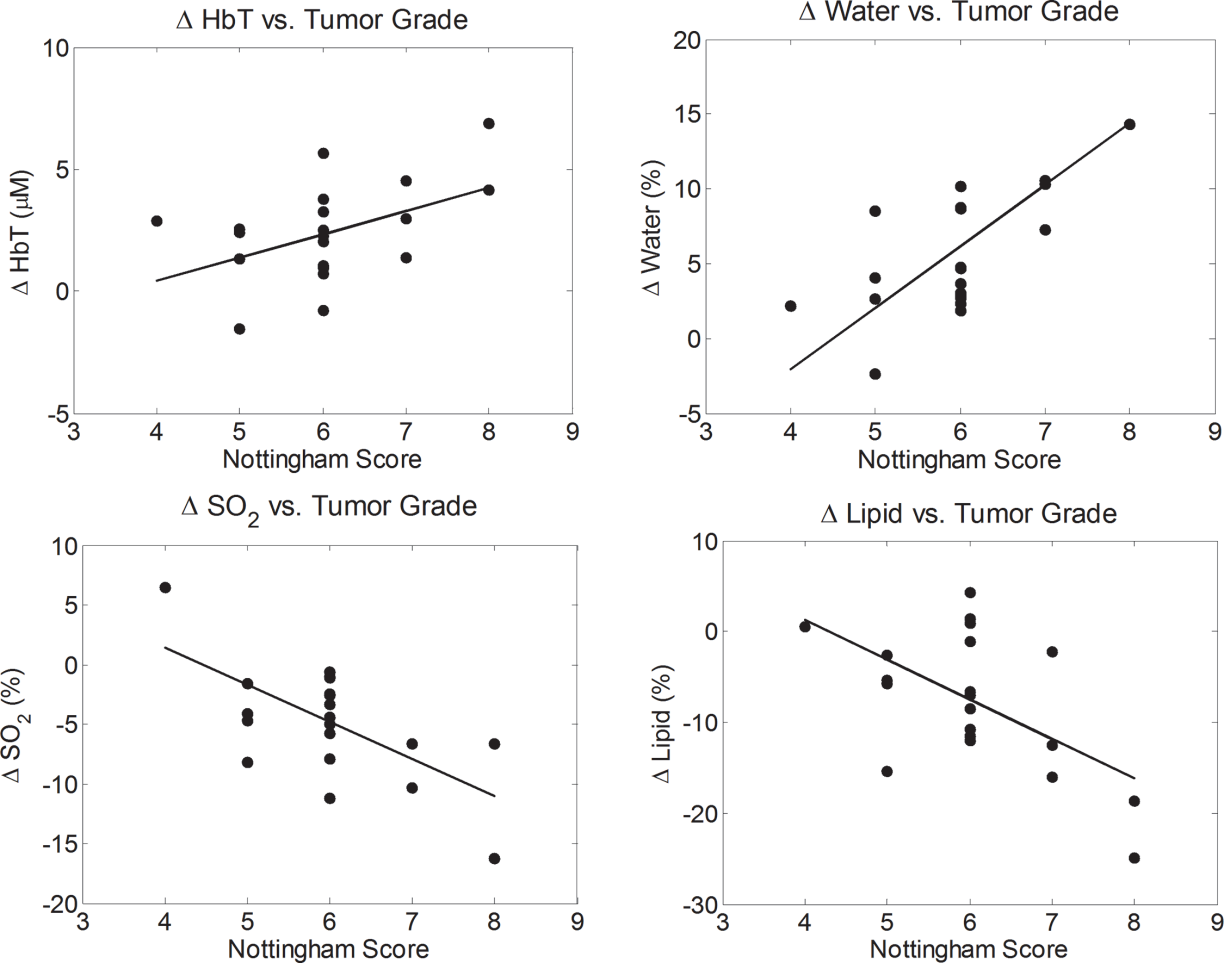
Difference quantities in the total hemoglobin concentration, water concentration, lipid concentration, and hemoglobin saturation plotted against the Nottingham histologic score. A linear trend showing the higher Nottingham Score corresponding to an increase in the magnitude of the difference between the tumor region and background tissue for [HbT], [water], [lipid], and SO_2_.

The difference parameters of the cancerous breasts were plotted against the Nottingham histologic score, the size of the tumor, and the HER2/neu receptor status. The over-expression of human epidermal growth factor receptor 2, HER2/neu, is associated with a poor prognosis of breast cancer. No significant correlation was observed between the difference parameters and the size of the tumor or the HER2/neu receptor status for this patient population. However, the Nottingham score tended to increase with the size of the tumor where a linear fit was found to have a *p* value of 0.057, which is associated with the significance of the slope value versus a null hypothesis of zero slope. Significant trends were found between the optically measured difference parameters and the Nottingham histologic score. The Nottingham score is a measure of intrinsic biologic aggressiveness and serves to both predict outcome and determine the course of treatment for a given patient. The difference parameters in [HbT], [water], [lipid], and SO_2_ were plotted against the Nottingham histologic score and, as seen in Fig. 7, the magnitudes of the difference parameters generally increase with a higher score of breast cancer. A linear fit was obtained for each plot and the slopes, errors, and *p*-values were also determined. The [water] difference was found to have the strongest linear trend, with a slope of +4 ± 1% per unit Nottingham score (*p* = 0.0001). The difference in [HbT] had the weakest linear trend, with a slope of 1.0 ± 0.4 μM per unit Nottingham score (*p* = 0.0325). The slopes for the difference in SO_2_ and [lipid] plots were −3 ± 1% per unit Nottingham score (*p* = 0.0014) and −4 ± 1% per unit Nottingham score (*p* = 0.0083), respectively. Similar trends between the water concentration in breast tumors and the histologic score have been previously reported [1]. These results indicate that our optical imaging system provides breast cancer measurements that correlate with a histologic assessment of tumor grade.

## Discussion

### The potential value of breast cancer oximetry

In this study, we have reported that breast tumors have a higher [HbT] and [water], and a lower [lipid] compared to the background tissue within the same breast. These results are in agreement with previous reports in the field where similar trends have been found in the concentration of hemoglobin [1–11], water [1,6,9–11], and lipids [1,11] in breast cancer. We have also found that breast tumors have significantly lower hemoglobin saturation than background tissue which may reflect the hypoxic conditions measured in tumor microenvironments [14–16,18,19]. Because cancer-induced effects on the oxygen metabolic rate (associated with changes in SO_2_) may occur earlier than angiogenic, structural, and biochemical alterations (associated with hemoglobin, water, and lipid concentration changes), hemoglobin saturation measurements may provide early indications of carcinogenesis. Furthermore, SO_2_ measurements, which indicate the balance between oxygen supply and oxygen consumption in tissue, provide a different source of contrast than that offered by measurements of hemoglobin concentration (which reflect levels of vascularization), and water or lipid content (which reflect biochemical and structural tissue composition). As a metabolic indicator, SO_2_ measurements can effectively complement the information provided by the hemoglobin, water, and lipid content in tissue which enhances the diagnostic and monitoring capabilities of optical mammography for breast cancer.

There is value in using optical imaging to track patient response to neoadjuvant therapy since the treated tumors are often large in size and *a priori* information is available to localize the cancer within the breast. Tissue hypoxia can convey information about the likelihood of a tumor’s resistance to chemotherapy or radiation [38], and it has been found in a NIRS study that baseline SO_2_ measurements correlate to patients’ response to neoadjuvant therapy [39]. Accurately quantifying the SO_2_ values in cancerous breast tissue has the potential to achieve clinical value by providing information to help determine the most effective therapeutic regimens and the anticipated individual response to neoadjuvant breast cancer therapy.

### Is hemoglobin saturation a reliable source of contrast in breast cancer?

Our findings of a lower SO_2_ in breast cancer confirm some previous NIRS studies [7, 9–10, 21], yet are at odds with a number of others that have not found breast tumors to feature a significant reduction in SO_2_ [1,2,5,6,8,11]. While we are unable to conclusively pinpoint the reasons for the mixed results reported in the literature, we can identify some potential factors to consider.

The spectral information from various optical measurements (a few discrete wavelengths, a continuous spectral band, the inclusion of wavelengths above 900 nm, etc.) can have an impact on the robustness of the chromophore concentration recovery, including oxy-hemoglobin and deoxy-hemoglobin. The accuracy, precision, and robustness of various measurements may be affected by source-detector geometries (such as transmission in a planar configuration or circular arrangement, diffuse reflection, etc.), the methods used to determine chromophore concentrations and hemoglobin saturation (diffusion theory for homogeneous media, perturbation approaches, etc.), and the various imaging techniques (simple backprojection, tomographic reconstruction schemes, etc.). Different system configurations may also impact contrast levels found (for all optical parameters, including SO_2_), especially when comparing homogeneous models versus perturbation approaches. For example, one would typically expect a larger tumor to create a greater optical contrast. However, our imaging technique is a 2D approach using a homogenous model and consequently the depth of the tumor plays a role in our optical contrast found. Therefore, the size of the tumor may be further confounded by its depth within the breast resulting in no significant relationship between the size and any of the optically measured difference parameters in this work.

Also, there are several possible choices for where to consider the healthy reference tissue. In this work, we chose the global background in the ipsilateral breast (i.e. the cancerous breast), with the exclusion of the cancer area as our reference tissue. This choice was made to minimize the variability associated with measurements on different breasts of the same patient (*i*.*e*. the contralateral breast), however, other studies have used the contralateral breast as the reference tissue [1,10]. To investigate the effect of this choice on our own results, we have re-analyzed our data by selecting the reference area to be the tissue on the contralateral breast that mirrors the location of the tumor in the cancerous breast. With this choice of reference tissue, we ran the same statistical test described in the methods section (Wilcoxon signed rank test) to determine if there was a significant difference in the optically measured quantities. We found that for [HbT] and [lipid], the *p* values were 0.05 and 0.002, respectively. Both *p* values were larger than those found when considering reference tissue in the ipsilateral breast, but still indicate significantly higher [HbT] and [lipid] in the tumor. As for SO_2_ and [water], the *p* values were found to be 0.12 and 0.07, respectively, implying no significant difference between the cancerous region and the reference tissue in the contralateral breast. Choosing healthy tissue in the contralateral breast as a reference introduces the additional variability associated with a different breast which may have biological as well as instrumental contributions. In summary, due to a range of characteristics across different studies and a potential intrinsic variability over cancer types and stages, the significance of SO_2_ as a source of contrast in breast cancer remains an open question in the NIRS field.

### Absolute vs. relative measurements

When characterizing how optically measured quantities change in breast tumors, studies have reported either absolute measurements on group averages of cancerous and healthy tissues [1,6,7,26] or relative measurements between cancerous and healthy tissues [2,6,11]. If the difference between the measurements on cancerous and healthy tissue dominates the inter-patient variability, then group averages of absolute measurements offer the ability to establish a cutoff value (in terms of chromophore concentrations, SO_2_ levels, or any other optically measured quantity) for the identification of cancer. However, such “absolute characterization” may be impacted by the inter-subject variabilty and non-uniformity in the optical properties of different breasts when averaging these values across a patient population. Different measured quantities may be affected differently by inter-patient variability and therefore be more or less suited for absolute comparisons. In our study, we have focused on the differences between tumor regions and healthy background tissue because the accuracy of our absolute measurements for each patient was affected by the assumed fixed values for the scattering parameters (μ_s0_ˊ and *b*). Therefore, the error in the absolute chromophore concentrations may vary from patient to patient depending on how close our assumed scattering properties were to the true values for each individual case. To further characterize and quantify this error, we considered a cancer patient dataset (#2) and performed the inverse model fits four additional times using the standard deviations reported in the literature to account for the possible range of values for the scattering parameters. We ran fits by using: μ_s0_ˊ(650 nm) = 10.8 ± 1.4 cm^−1^ and *b* = 1.0 ± 0.4 [5]. Despite the absolute concentrations of the background or tumor changing by up to 40%, the sign of the difference (tumor-background) remained the same for all quantities considered (higher [HbT] and [water] in the tumor; lower SO_2_ and [lipid] in the tumor). The magnitude of the difference parameters also varied minimally, never exceeding the range of the mean ± standard deviation as reported in Fig. 6 for the various quantities. On this point, we also observed that some optical studies using time domain or frequency domain systems have reported a scattering contrast between tumors and healthy tissue [1,2,4,9,11] which we have not taken into account in this study.

### Population means vs. individual patients

We have measured tumor-background differences for [HbO], [Hb], [HbT], [water], [lipids], and SO_2_ and after taking the mean over the 26 patients, each of these difference parameters has been found to be significantly different than zero. It is important to note, however, that tumor-background differences for the chromophore and SO_2_ quantities within each individual patient were not always found to be significantly different than zero. A two-sample *t* test was carried out for the statistical analysis of the individual patient data. The *t* test should take into account the number of degrees of freedom of the measurements. Because of the significant correlation between the pixels in diffuse optical data collected by the source and detector every 2 mm (our scanning step), the number of degrees of freedom is not simply given by the number of measurements in the tumor or reference areas. In efforts to find the measurement degrees of freedom, we used the analytical description of the region of optical sensitivity (the “banana” region) [40] with typical optical properties of breast tissue. We then determined the maximum width of the 75% region of sensitivity (i.e. the “banana” containing voxels to which optical measurements have a sensitivity of at least 75% that of the maximal value on the geometrical source-detector line) for the individual source-detector separation recorded for each patient (which was in the range 4.3–9.2 cm). This resulted in a spatial down-sampling from 2 mm per data point to 10–18 mm per uncorrelated data point, which provided our estimate for the number of degrees of freedom for each patient.

**Fig 8:**
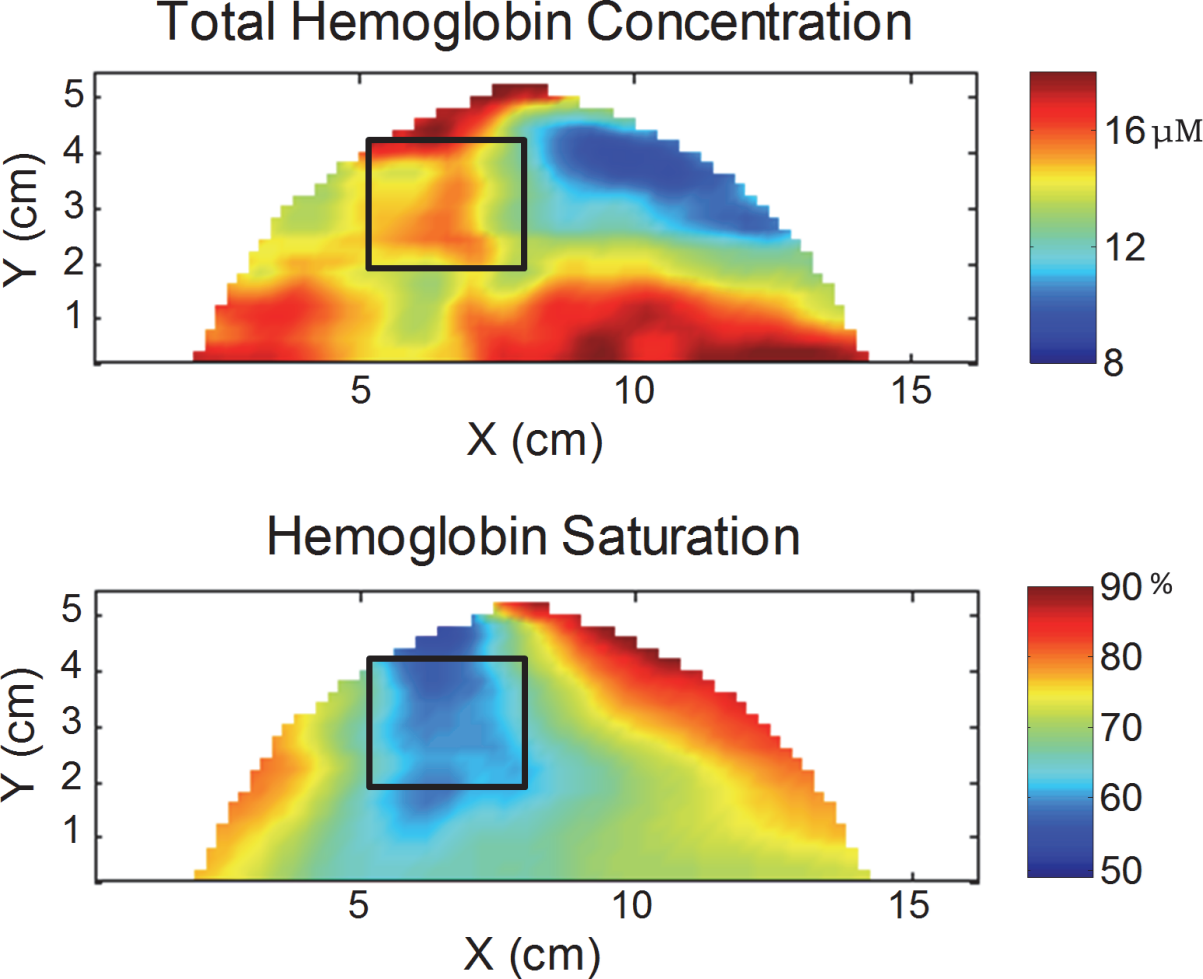
Optical maps of [HbT] and SO_2_ for a 59 year old patient with invasive ductal carcinoma in her left breast. The black box indicates the tumor region identified based off of findings from the x-ray mammogram. The total hemoglobin concentration maps show a local increase in the region on the tumor, yet other areas of the breast also have increased [HbT]. The saturation maps portray that only in the tumor region is there a decrease in SO_2_.

Two-sample *t* tests on individual patients showed that 8 out of 26 cancer cases did not show a significantly different amount of [HbT] in the tumors when compared to the background. In 4 of these cases, local increases in total hemoglobin can still be seen in the spatial maps, so that a choice of healthy reference tissue restricted to regions closer to the cancer would still result in a significantly higher [HbT] in the tumor. These 4 cases, however, did show a significantly lower SO_2_ in the tumor, confirming the different source of contrast from [HbT] and SO_2_. In Fig. 8 the [HbT] and SO_2_ maps are shown for patient #21, where the local increase in [HbT] is seen in the breast (highlighted by the black box) and the corresponding decrease in SO_2_ can also be observed. In the other 4 cases where the tumor [HbT] was not found to be significantly different than in the remainder of the breast, the tumor SO_2_ was also found not to be significantly different from reference tissue. These results indicate some intrinsic limitations of optical mammography in the detection of individual tumors, and they also suggest that the additional information of SO_2_ may help identify some low-contrast tumors.

## Conclusion

This optical mammography study has characterized cancer-induced changes in chromophore concentrations (hemoglobin, water, and lipids) and oxygen saturation of hemoglobin (SO_2_) in 26 breast cancer patients. A key finding of this work is the significant reduction of SO_2_ in breast cancer, a result that may be used to enhance the information content of optical mammograms. In fact, SO_2_ measurements may provide sensitivity to early stage metabolic changes in cancer and serve as a valuable marker for tumor regression during neoadjuvant chemotherapy.
